# Efficacy and Biomarker Exploration of Sintilimab Combined With Chemotherapy in the Treatment of Advanced Penile Squamous Cell Carcinoma—A Report of Two Cases

**DOI:** 10.3389/fonc.2022.823459

**Published:** 2022-04-07

**Authors:** Xinkuan Mei, Yanyan Zhao, Yiruo Zhang, Jinhua Liao, Chen Jiang, Hesheng Qian, Yingying Du

**Affiliations:** ^1^Department of Oncology, Fuyang Tumor Hospital, Fuyang, China; ^2^Department of Oncology, First Affiliated Hospital of Anhui Medical University, Hefei, China

**Keywords:** penile cancer, immune checkpoint inhibitors, biomarkers, tumor immune microenvironment, case report

## Abstract

Penile squamous cell carcinoma is a rare malignant tumor of the male reproductive system. We report two cases of advanced penile squamous cell carcinoma with persistent partial response/complete response after sintilimab combined with chemotherapy and analyze the relevant tumor biomarkers.

## Introduction

Penile cancer is considered a rare malignancy, with an incidence of penile squamous cell carcinoma in the United States ranging from 0.1 to 1 per 10,0000 (1). Squamous cell carcinoma is the most common histological type, and its pathogenesis is closely related to chronic human papillomavirus (HPV) infection. Immunotherapy has made progress in many malignant tumors, especially in squamous cell carcinomas, such as head and neck squamous cell carcinoma, lung squamous cell carcinoma, and esophageal squamous cell carcinoma. The predictors of efficacy in immunotherapy for these squamous cell carcinomas are unclear. The two most commonly used biomarkers are programmed death ligand-1 (PD-L1) expression and tumor mutation burden (TMB); however, neither PD-L1 expression nor TMB is a definite biomarker for immune therapy prediction in squamous cell carcinoma, as responses are also observed in PD-L1-negative and low TMB patients (2). In penile squamous cell carcinoma, the expression of PD-L1 has been reported to range from 40% to 62% (3). Studies have shown that PD-L1 expression is independent of patient age, tumor location, histological subtype, tumor stage, anatomical depth of tumor invasion, and tumor grade in patients with advanced penile squamous cell carcinoma (4). HPV-negative tumors have a significant proportion of PD-L1 expression compared with HPV-positive tumors (5). Tumor PD-L1 expression is significantly associated with lymph node metastasis. Tumors with diffuse positivity for PD-L1 show a higher probability of lymph node metastasis than tumors with a marginal expression of PD-L1 or tumors with negative expression of PD-L1, present a higher risk of disease-specific death (6, 7). High expression of PD-L1 is associated with short survival, so it can be used as a factor to identify a poor prognosis in penile cancer (3). TMB is defined as the total number of somatic/acquired mutations per coding area of a tumor genome; in penile cancer, TMB values between 3.6 and 4.5 mutations/Mb have been reported (5).

Immunotherapy for penile squamous cell carcinoma is currently under clinical study; some results showed that immunotherapy could effectively improve the disease control and prognosis (8, 9). Sintilimab is an immune checkpoint inhibitor that acts on programmed cell death-1 (PD-1). It has been confirmed to have a good efficacy in a variety of advanced tumors, such as lung cancer, esophageal cancer, and gastric cancer. Here, we report two patients with advanced penile squamous cell carcinoma who were administered chemotherapy combined with sintilimab and discuss associated tumor biomarkers.

## Case Reports

Patient A, a 63-year-old man, was diagnosed with well-differentiated squamous cell carcinoma of the penis in February 2014. He received partial penectomy without postoperative chemoradiotherapy. In December 2018, the patient presented a local recurrence of penile cancer and received a further partial penectomy. The postoperative pathology showed moderate to poorly differentiated squamous cell carcinoma of the penis invading the corpus cavernosum. The patient did not receive chemoradiotherapy after surgery. In November 2019, lung metastases were found, and the TNM stage of which was T3NxM0. From November 2019 to March 2020, the patient received paclitaxel 180 mg + nedaplatin 120 mg + sintilimab 200 mg for 6 cycles, and the treatment efficacy indicated a partial response. From April 2020 to March 2021, the patient was given maintenance immunotherapy with sintilimab 200 mg for 1 year. The sustained partial response was achieved during this period. The drug was subsequently discontinued due to a grade III immune-related rash. The patient was followed up regularly and currently maintains a persistent partial response.

**Figure 1:**
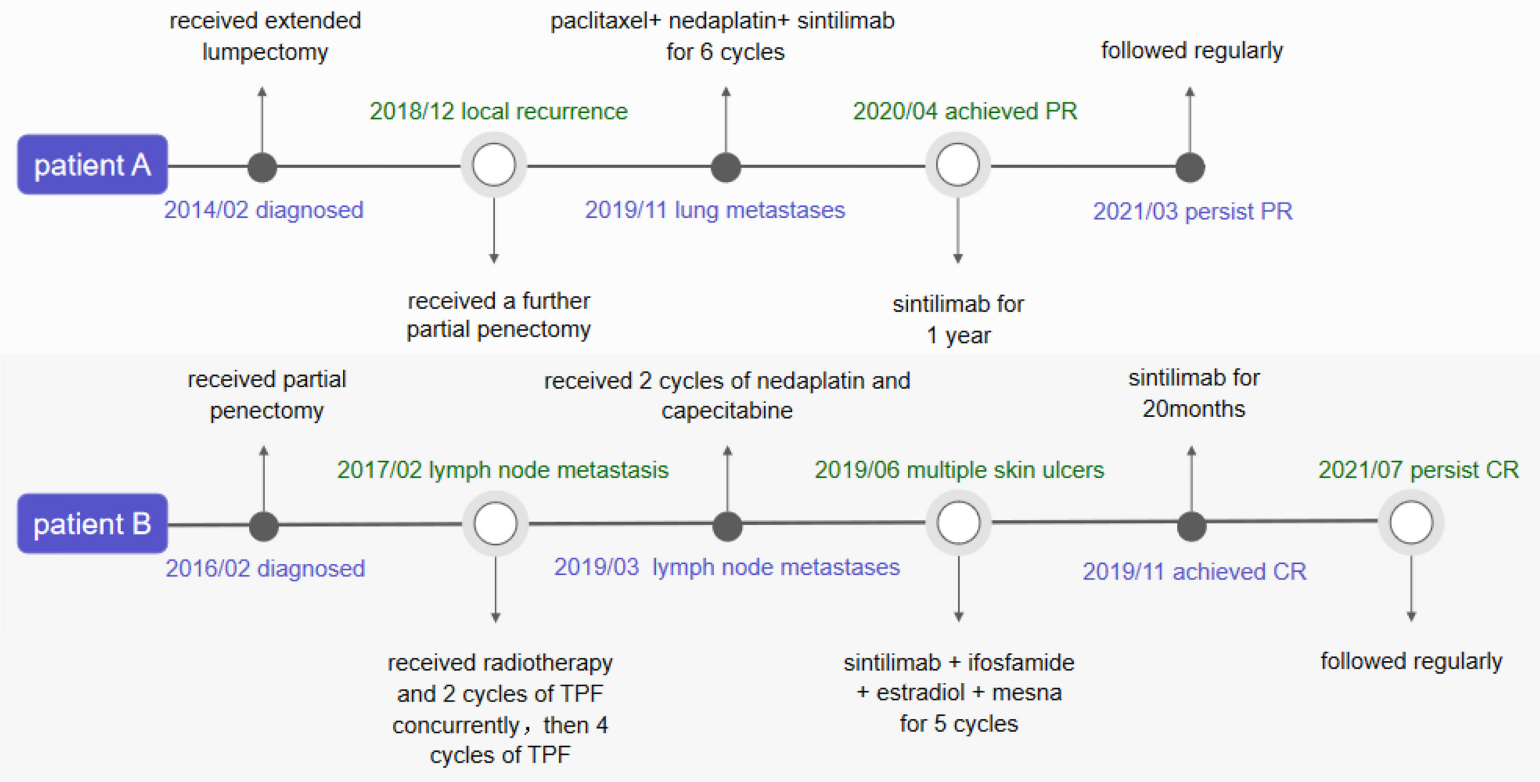
Timeline of the two patients**’**s therapy and effect of therapy.

**Figure 2:**
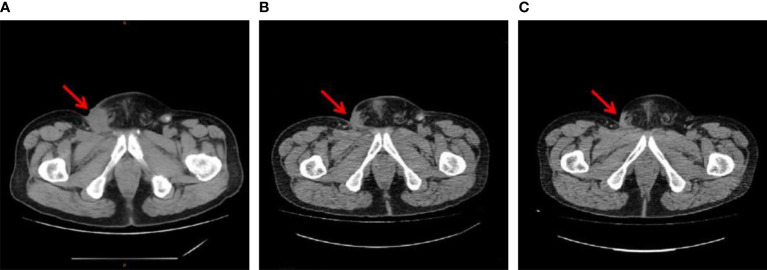
Inguinal metastasis imaging of patient B **(A)** before sintilimab plus chemotherapy (June 13, 2019), **(B)** 6 months after sintilimab plus chemotherapy (December 20 2019), and **(C)** 2 months after the end of sintilimab maintenance therapy (September 08, 2021).

Patient B, a 39-year-old man, underwent partial penectomy due to moderately differentiated squamous cell carcinoma of the penis in February 2016. The postoperative pathology revealed the tumor had invaded into the corpus cavernosum in the absence of chemoradiotherapy. In February 2017, the patient was found to have lymph node metastasis in the inguinal region, and the TNM stage of which was T3N3M0. He then received 60 GY/30 F three-dimensional modulated intensity radiotherapy for cavernous metastasis, bilateral inguinal, right pelvic enlarged lymph nodes, and corresponding lymphatic drainage area, and received concurrently, 2 cycles of TPF chemotherapy (docetaxel + cisplatin + tegafur). A partial response was achieved after 2 cycles, and 4 cycles of TPF chemotherapy were continued after the concurrent chemoradiotherapy. The patient achieved a progression-free survival of approximately 35.9 months after the first-line treatment. In March 2019, the patient’s lymph nodes in the inguinal region of the right radiation field continued to expand, and the puncture confirmed metastatic squamous cell carcinoma. The patient received 2 cycles of second-line chemotherapy with nedaplatin + capecitabine, and in June 2019, the disease progressed, although multiple ulcers on the skin of the right abdominal region were observed, accompanied by pain and exudation. From June 2019 to September 2019, the patient was treated with a third-line regimen of sintilimab 200 mg + ifosfamide 2 g + estradiol 40 mg + mesna 0.4 g for 5 cycles. A partial response was evaluated after 2 cycles, and a complete response was achieved after 4 cycles. From November 2019 to July 2021, the patient received maintenance immunotherapy with sintilimab 200 mg. Currently, the patient has completed a 2-year treatment with sintilimab. Treatment has been stopped, and the patient is being followed up regularly. The evaluation shows a sustained complete response (**Figures 1**, **2**).

## Tumor Biomarker Detection

Immunohistochemical examinations were performed using surgically excised specimens obtained from 2 patients. Patient A was wild-type p53, which is established as a potent tumor suppressor; whereas, patient B was mutant p53, which is often associated with tumor malignancy and therapy resistance. Both patients were P16 negative, HPV negative, and proficient for mismatch repair proteins (MMR proficient). P16 has been used as a surrogate marker for active HPV infection (10). MMR proficient and microsatellite stable have been shown in clinical studies for colorectal cancer to be associated with limited immunotherapy efficacy (11).

**Figure 3:**
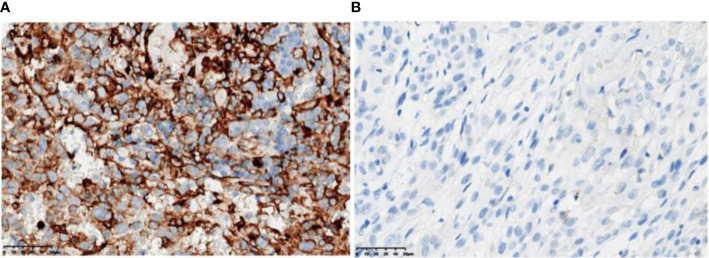
PD-L1 immunohistochemistry test (antibody model: SP263). **(A)** Patient A has a positive PD-L1 immunohistochemistry with 50% to 60% PD-L1-positive tumor cells (TC) and 15% PD-L1-positive tumor-associated immune cells (IC). **(B)** Patient B was negative for PD-L1 immunohistochemistry with <1% TC and 1% IC.

The entire exon regions of 310 genes and the hotspot mutation regions of 210 genes were evaluated by probe hybridization and high-throughput sequencing in primary tumor tissue samples from the two patients. TMB values were calculated, and microsatellite instability was detected. The VENTANA PD-L1 (SP263) assay (Roche) (Ventana Medical Systems, Inc., Tucson, AZ, USA) was used to determine the PD-L1 expression. Multiplex fluorescence immunohistochemistry was used to detect the expression of CD8, CD3, PD-L1, and PD-1 in tumor tissue samples. Mantra System (PerkinElmer, Waltham, MA, USA) was used for imaging studies. InForm image analysis software (Version 2.4, PerkinElmer, Waltham, MA, USA) was used for the identification of tumor tissues and cells. The density and percentage of positive cells with different markers were calculated in the tumor parenchyma and stroma, respectively, to evaluate the tumor immune microenvironment (**Figure 3** and **Table 1**).

**Table 1:** Detection of biomarkers in patients A and B.

Test items		Patient A	Patient B
Gene mutations with clinically significant		HRAS LRP1B TERT	Null
Microsatellite instability		Microsatellite stable	Microsatellite stable
TMB		17.95 mutations /Mb	0 mutations /Mb
PD - L1 expression			
PD-L1 positive tumor cells(TC)		50%-60%	<1%
PD-L1 positive tumor associated immune cells(IC)		15%	1%
Tumor Parenchyma			
CD8^+^T cells	Density	121 cells/mm^2^	201 cells/mm^2^
	Positive rate	1.70%	3.28%
CD3^+^T cells	Density	439 cells/mm^2^	315 cells/mm^2^
	Positive rate	6.19%	5.14%
PD-L1^+^T cells	Density	1268 cells/mm^2^	261 cells/mm^2^
	Positive rate	17.88%	4.26%
PD-1^+^T cells	Density	8 cells/mm^2^	18 cells/mm^2^
	Positive rate	0.12%	0.30%
Tumor Stroma			
CD8^+^T cells	Density	837 cells/mm^2^	801 cells/mm^2^
	Positive rate	8.06%	11.14%
CD3^+^T cells	Density	2599 cells/mm^2^	1455 cells/mm^2^
	Positive rate	25.02%	20.24%
PD-L1^+^T cells	Density	1302 cells/mm^2^	367 cells/mm^2^
	Positive rate	12.53%	5.10%
PD-1^+^T cells	Density	141 cells/mm^2^	95 cells/mm^2^
	Positive rate	1.36%	1.33%

No genetic mutations of definite clinical significance were detected in the two patients, but 3 genetic mutations with potential clinical significance were found in patient A. Studies of relevant tumor biological markers for the two patients showed that both were HPV negative, but the expression levels of PD-L1 and TMB levels were very different. The reality is that both are effective for immunotherapy and show sustained partial response/complete response. By analyzing the tumor immune microenvironment (TIME), it is found that both TIME have high expressions of CD3^+^/CD8^+^ cells in tumor parenchyma and tumor stroma.

## Discussion

The TIME is a key target for immunotherapy in cancer patients. The expression of tumor-related immune markers to define the TIME can be divided into subtypes according to the expression levels of PD-L1 and the presence of tumor-infiltrating lymphocytes (TIL): immune neglect type (B7^−^H1^−^/TIL^−^), adaptive immune tolerance type (B7^-^H1^+^/TIL^+^), other pathway-dependent immune evasion types (B7^−^H1^−^/TIL^+^), and primary induced expression type (B7^−^H1^+^/TIL^−^) (12). Immunotherapy is most likely to be effective in the presence of TILs and B7-H1 expression. Little is known about the use of TIME in patients with penile cancer. In this study, it is found that the TIME of both is considered an adaptive immune tolerance type, which may provide explain the good efficacy of immunotherapy. We speculate that the presence of TILs in penile squamous cell carcinoma can be used as potent predictive biomarkers for the efficacy of immunotherapy, so TIME can be used to develop immunotherapeutic targets or evaluate whether immunotherapy is effective. These are just two cases, but since penile cancer is a rare tumor, further research can be carried out based on this study’s result.

In head and neck squamous cell carcinoma, a higher degree of T-cell infiltration was found in HPV-positive tumors compared with HPV-negative tumors, and involved CD8^+^ T cells within the tumor and stroma (13), with an increased rate of CD8^+^ T-cell infiltration in the tumor-associated stroma being significantly associated with lymph node metastasis. HPV-positive tumors are characterized by a rich TIME, which has a better prognosis. Furthermore, patients with positive PD-L1 expression in tumor cells and higher levels of CD8^+^ TILs presented a shorter cancer-specific survival (14). In patients with penile squamous cell carcinoma, the relationship between HPV infection status, PD-L1 expression, and the TIME warrants further investigation and may help predict immunotherapy efficacy and screening effective populations.

Currently, clinical studies are being conducted to evaluate the use of immune checkpoint inhibitors in advanced penile cancer, and the exploration of biomarkers is also a focus of intense research. Our findings relative to the detection of tumor molecular markers and successful treatment of two patients with penile squamous cell carcinoma may provide new insights.

## Data Availability Statement

The original contributions presented in the study are included in the article/**Supplementary Material**. Further inquiries can be directed to the corresponding authors.

## Ethics Statement

The studies involving human participants were reviewed and approved by the Ethics Committee of Fuyang Cancer Hospital. The patients/participants provided their written informed consent to participate in this study.

## Author Contributions

XM and YYZ took the lead in drafting the manuscript and provided tumor biomarker detection data. YRZ, JL, and CJ were involved in drafting the manuscript. YD and HQ provided supervision and participated in the literature review. All authors read and approved the final manuscript.

## Conflict of Interest

The authors declare that the research was conducted in the absence of any commercial or financial relationships that could be construed as a potential conflict of interest.

## Publisher’s Note

All claims expressed in this article are solely those of the authors and do not necessarily represent those of their affiliated organizations, or those of the publisher, the editors and the reviewers. Any product that may be evaluated in this article, or claim that may be made by its manufacturer, is not guaranteed or endorsed by the publisher.
