# Supplementary material for: Efficacy and Biomarker Exploration of Sintilimab Combined With Chemotherapy in the Treatment of Advanced Penile Squamous Cell Carcinoma—A Report of Two Cases
Source: Front Oncol. 2022 Apr 7;12:823459. doi: 10.3389/fonc.2022.823459 (PMC9021724; doi:10.3389/fonc.2022.823459)
Supplement: Supplementary file 1 [file DataSheet_1.docx]

Supplementary Material

# Supplementary Figures and Tables

## Supplementary Figures

##
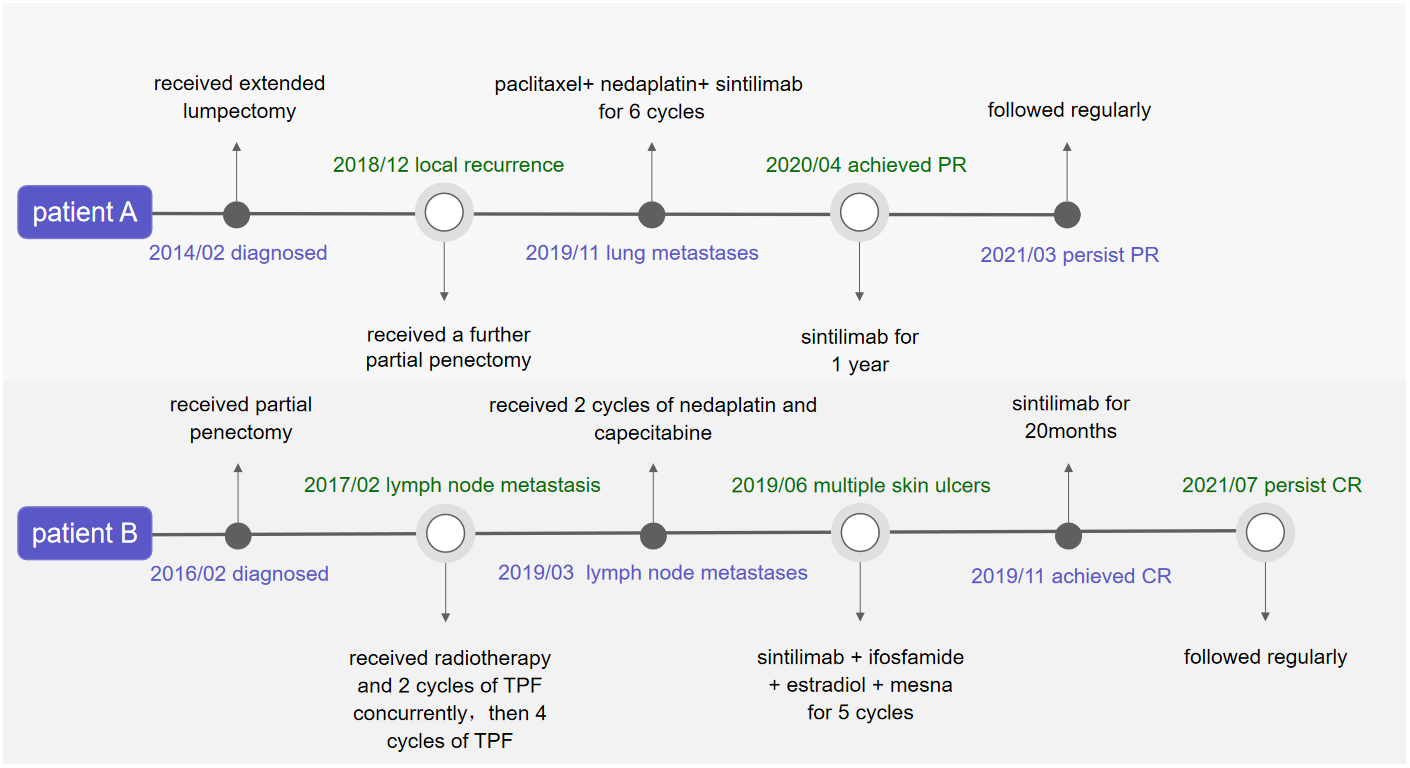


## Supplementary Figure 1. Timeline of the two patients’ s therapy and effect of therapy

##
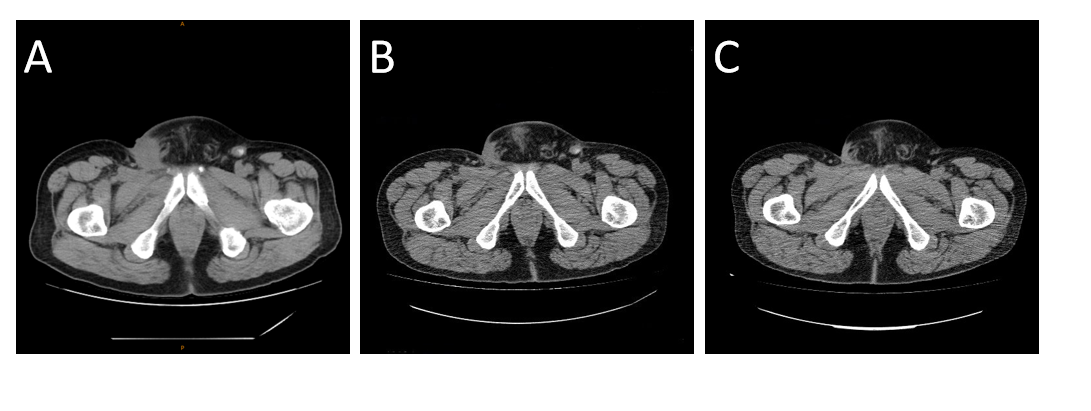


**Supplementary Figure 2.** Inguinal metastasis imaging of patient B,(A)before sintilimab plus chemotherapy (13 June 2019);(B)six months after sintilimab plus chemotherapy(20 December 2019);(C)2 months after the end of sintilimab maintenance therapy(08 September 2021).

##
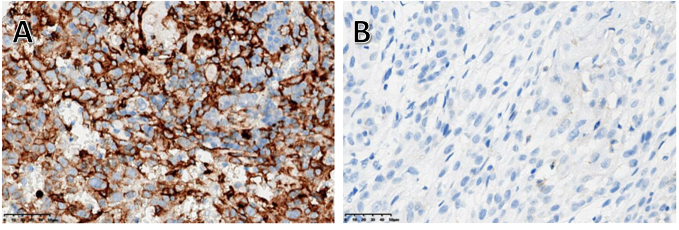


**Supplementary Figure 3.** PD-L1 immunohistochemistry test (antibody model: SP263) , (A)patient A has a positive PD-L1 immunohistochemistry with 50% to 60% PD-L1 positive tumor cells (TC) and 15% PD-L1 positive tumor associated immune cells (IC). (B)patient B was negative for PD-L1 immunohistochemistry with < 1% TC and 1% IC.

## Supplementary Table


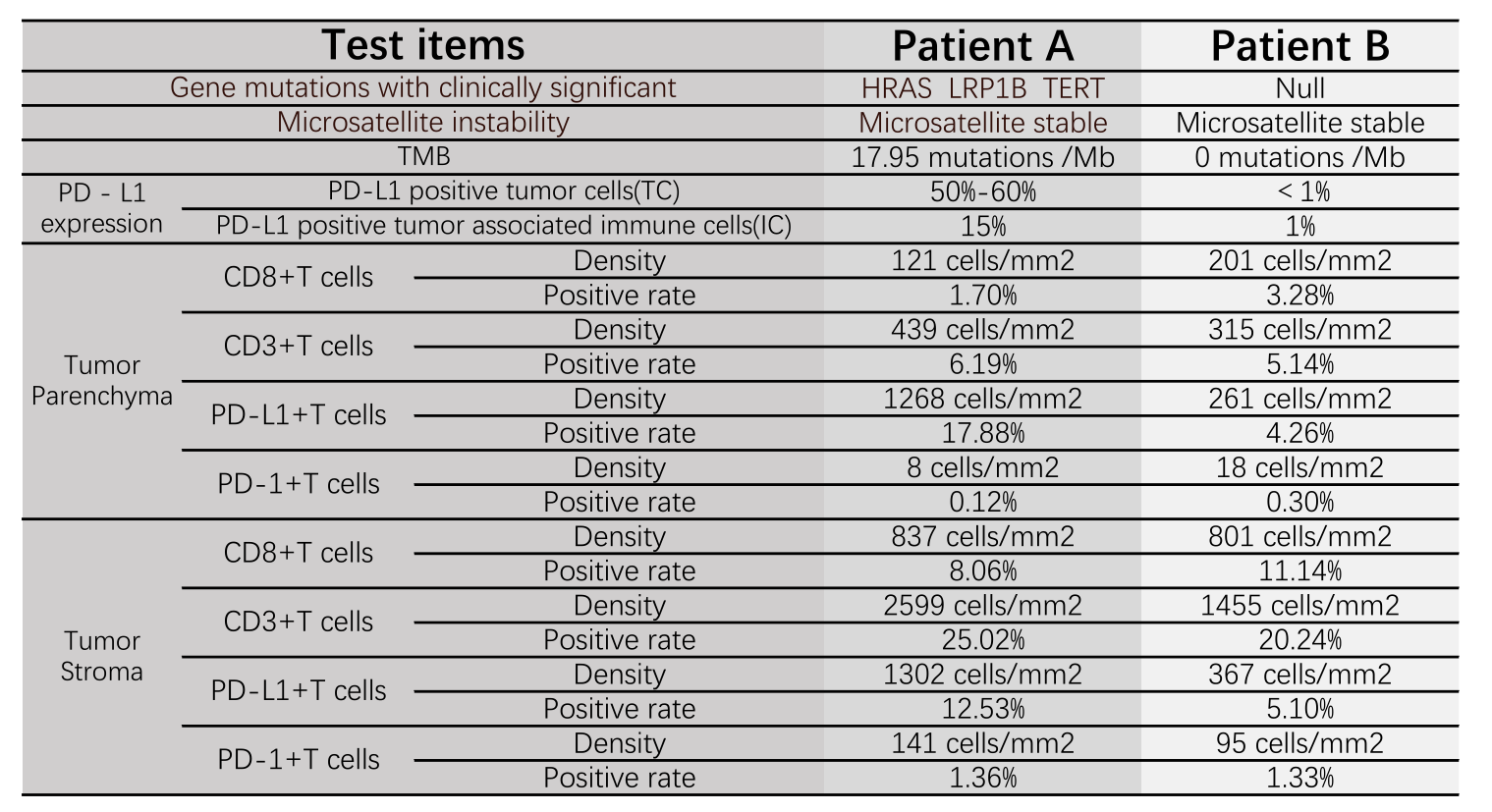


**Supplementary Table 1.** Detection of biomarkers in patients A and B

We uploaded the imaging of patient A’s lung metastases before and after immunochemotherapy as attachments.

A B C


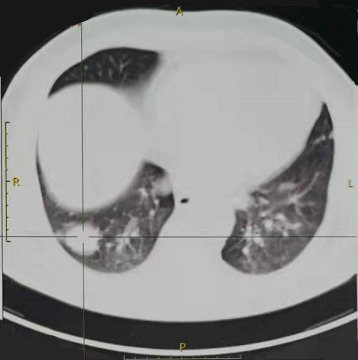

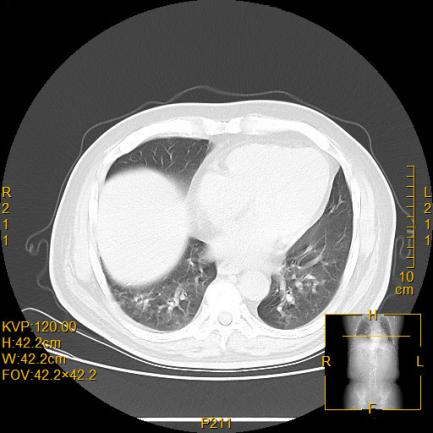

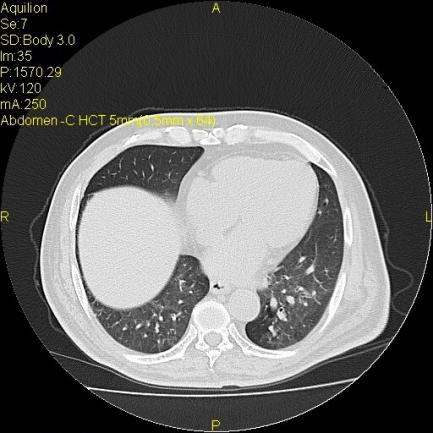


(A)Patient A lung metastasis before sintilimab plus chemotherapy (PET-CT)(20 November 2019)

(B)Patient A two months after sintilimab plus chemotherapy(06 January 2020)

(C)Patient A 5 months after the end of sintilimab maintenance therapy(16 November 2021)
